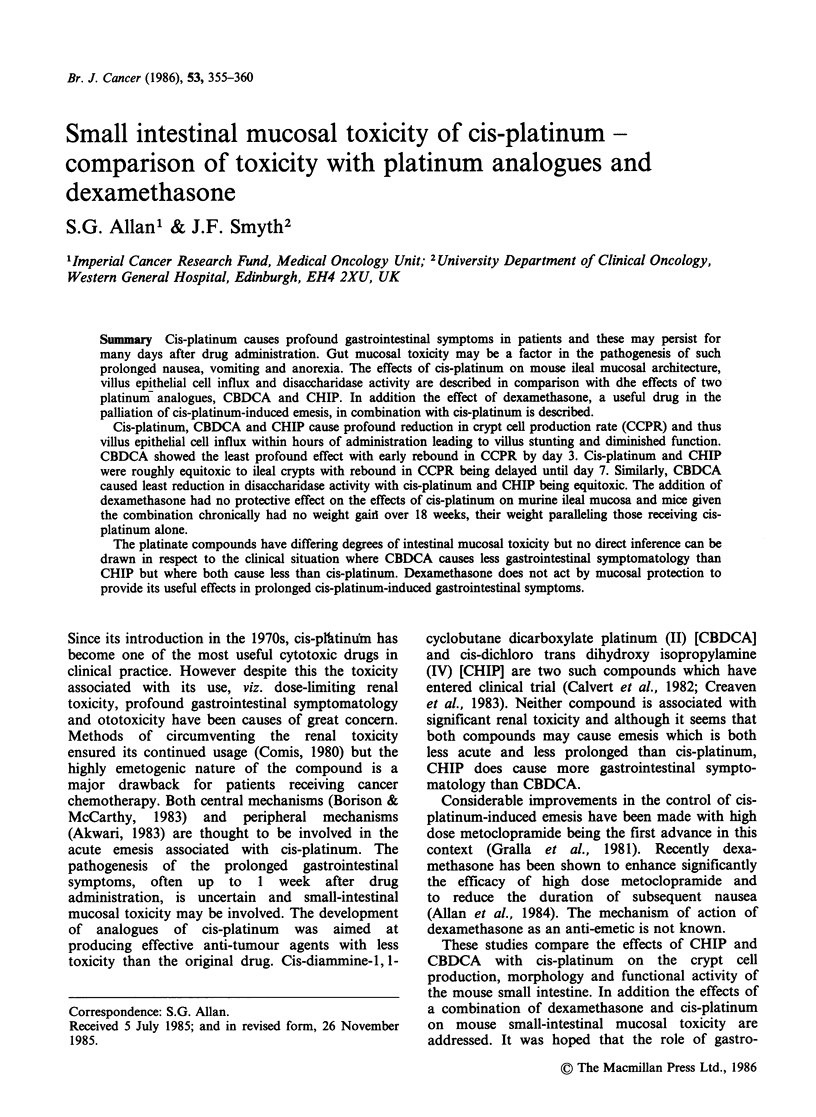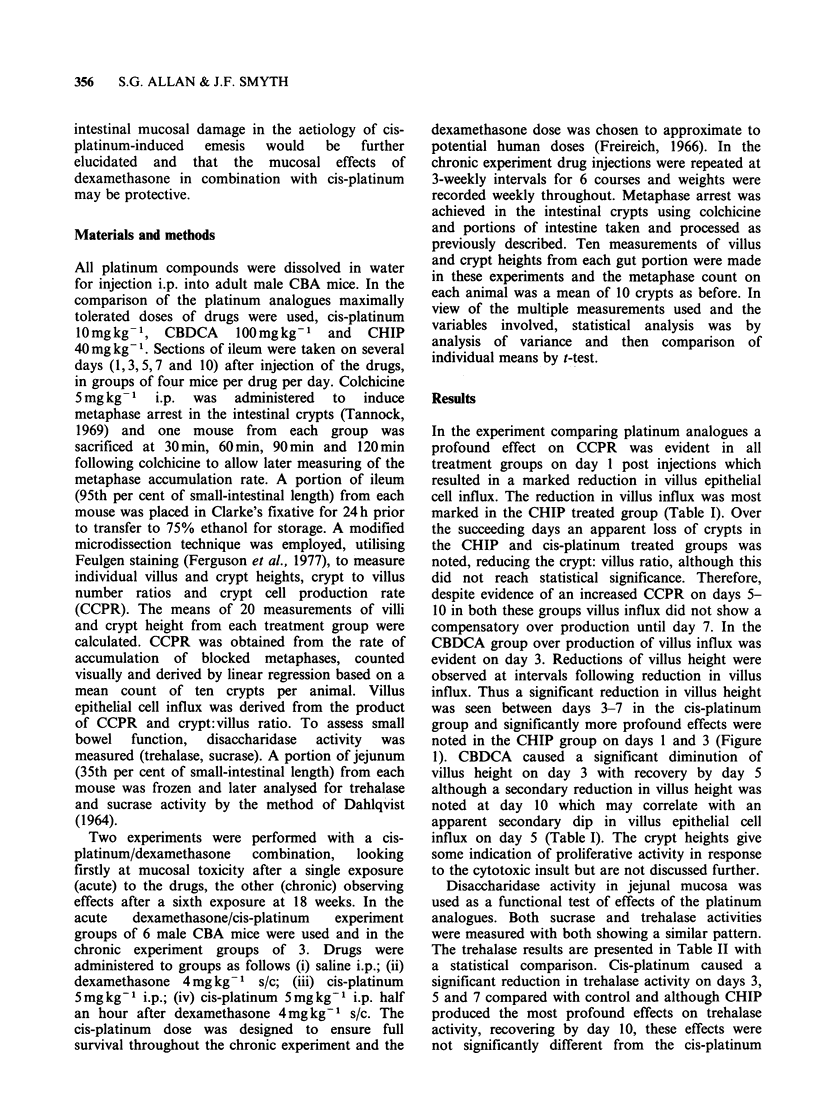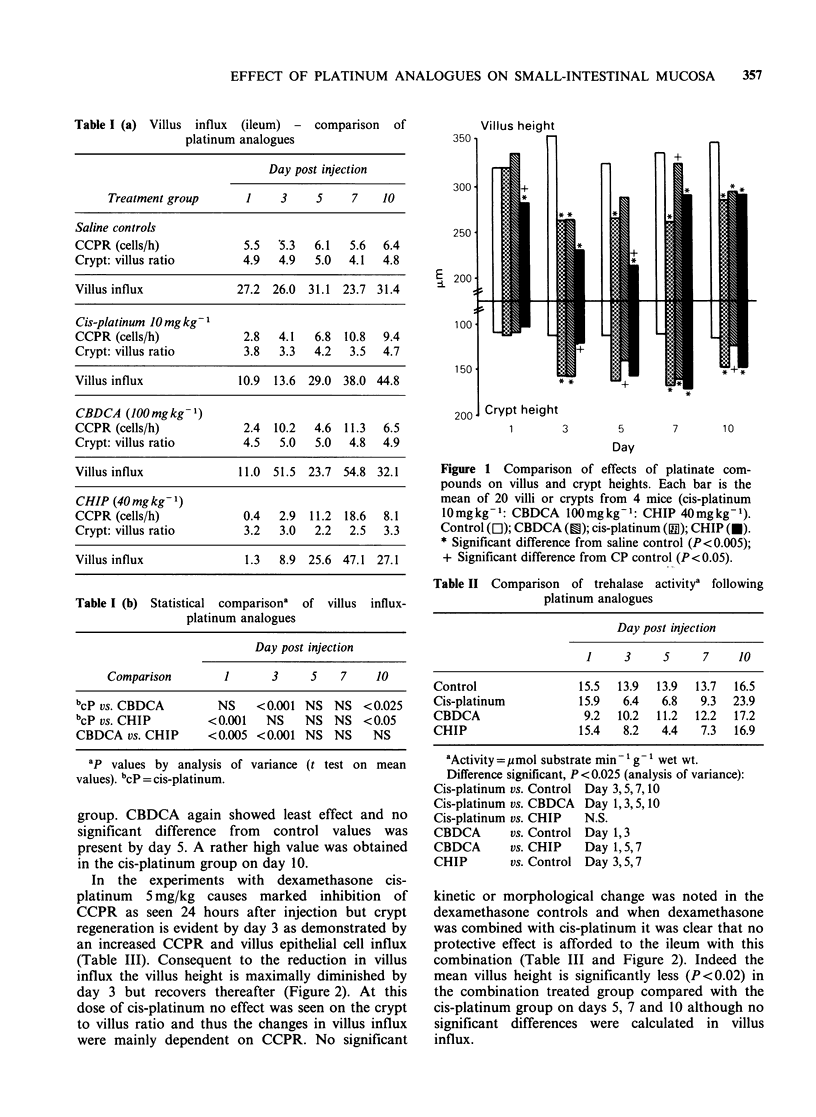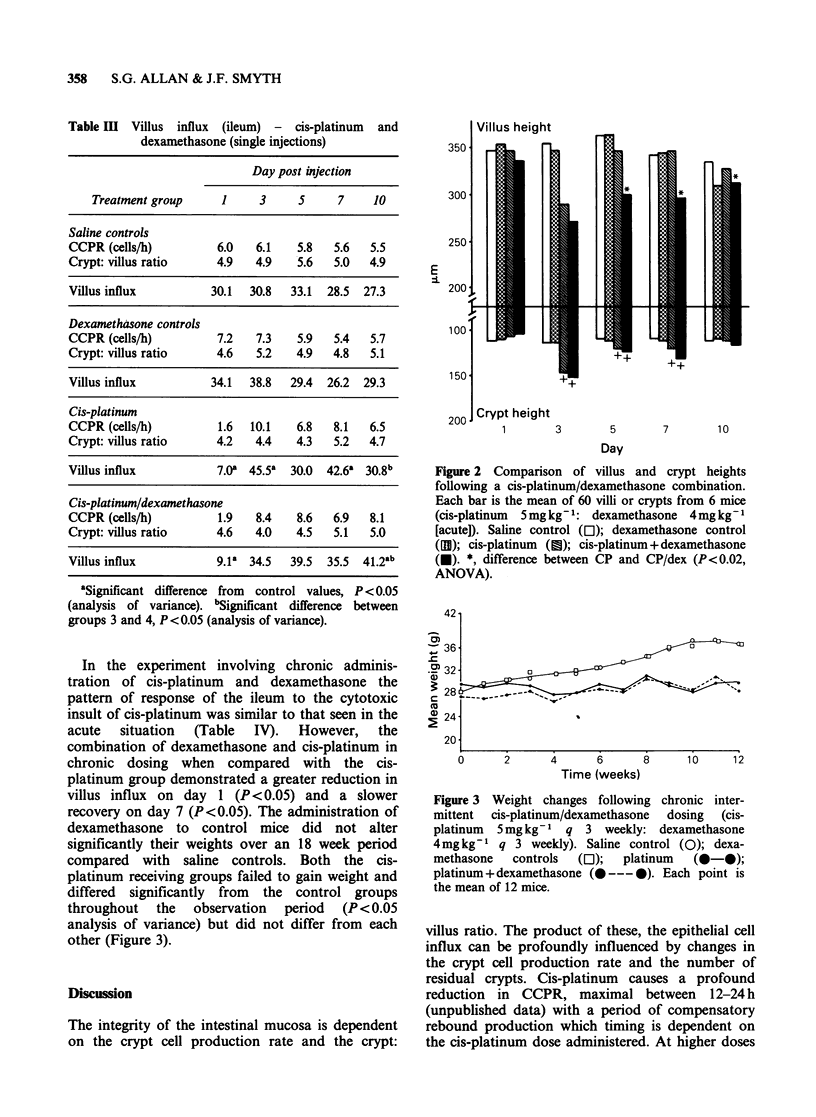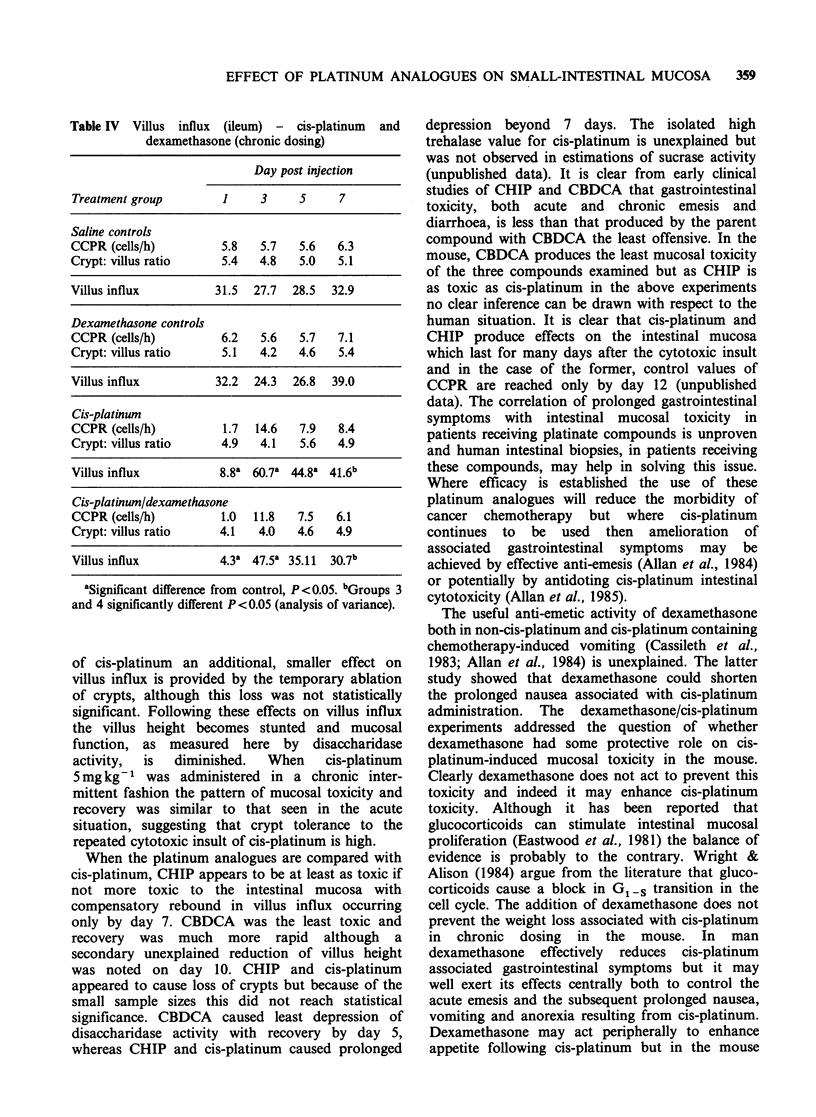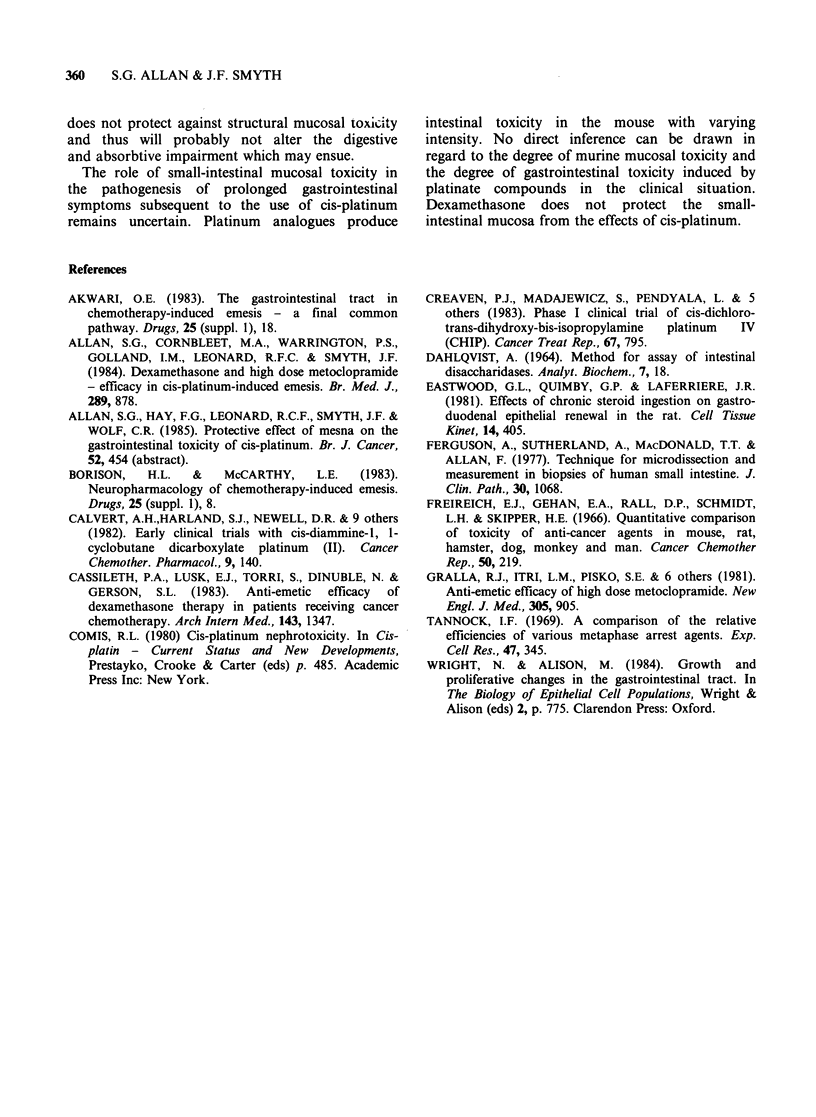# Small intestinal mucosal toxicity of cis-platinum--comparison of toxicity with platinum analogues and dexamethasone.

**DOI:** 10.1038/bjc.1986.59

**Published:** 1986-03

**Authors:** S. G. Allan, J. F. Smyth

## Abstract

Cis-platinum causes profound gastrointestinal symptoms in patients and these may persist for many days after drug administration. Gut mucosal toxicity may be a factor in the pathogenesis of such prolonged nausea, vomiting and anorexia. The effects of cis-platinum on mouse ileal mucosal architecture, villus epithelial cell influx and disaccharidase activity are described in comparison with dhe effects of two platinum analogues, CBDCA and CHIP. In addition the effect of dexamethasone, a useful drug in the palliation of cis-platinum-induced emesis, in combination with cis-platinum is described. Cis-platinum, CBDCA and CHIP cause profound reduction in crypt cell production rate (CCPR) and thus villus epithelial cell influx within hours of administration leading to villus stunting and diminished function. CBDCA showed the least profound effect with early rebound in CCPR by day 3. Cis-platinum and CHIP were roughly equitoxic to ileal crypts with rebound in CCPR being delayed until day 7. Similarly, CBDCA caused least reduction in disaccharidase activity with cis-platinum and CHIP being equitoxic. The addition of dexamethasone had no protective effect on the effects of cis-platinum on murine ileal mucosa and mice given the combination chronically had no weight gain over 18 weeks, their weight paralleling those receiving cis-platinum alone. The platinate compounds have differing degrees of intestinal mucosal toxicity but no direct inference can be drawn in respect to the clinical situation where CBDCA causes less gastrointestinal symptomatology than CHIP but where both cause less than cis-platinum. Dexamethasone does not act by mucosal protection to provide its useful effects in prolonged cis-platinum-induced gastrointestinal symptoms.


Br. J. Cancer (1986), 53, 355–360

### Small intestinal mucosal toxicity of cis-platinum – comparison of toxicity with platinum analogues and dexamethasone

S.G. Allan[1] & J.F. Smyth[2]

[1]*Imperial Cancer Research Fund, Medical Oncology Unit;* [2]*University Department of Clinical Oncology, Western General Hospital, Edinburgh, EH4 2XU, UK*

**Summary** Cis-platinum causes profound gastrointestinal symptoms in patients and these may persist for many days after drug administration. Gut mucosal toxicity may be a factor in the pathogenesis of such prolonged nausea, vomiting and anorexia. The effects of cis-platinum on mouse ileal mucosal architecture, villus epithelial cell influx and disaccharidase activity are described in comparison with dhe effects of two platinum analogues, CBDCA and CHIP. In addition the effect of dexamethasone, a useful drug in the palliation of cis-platinum-induced emesis, in combination with cis-platinum is described.

Cis-platinum, CBDCA and CHIP cause profound reduction in crypt cell production rate (CCPR) and thus villus epithelial cell influx within hours of administration leading to villus stunting and diminished function. CBDCA showed the least profound effect with early rebound in CCPR by day 3. Cis-platinum and CHIP were roughly equitoxic to ileal crypts with rebound in CCPR being delayed until day 7. Similarly, CBDCA caused least reduction in disaccharidase activity with cis-platinum and CHIP being equitoxic. The addition of dexamethasone had no protective effect on the effects of cis-platinum on murine ileal mucosa and mice given the combination chronically had no weight gain over 18 weeks, their weight paralleling those receiving cis-platinum alone.

The platinate compounds have differing degrees of intestinal mucosal toxicity but no direct inference can be drawn in respect to the clinical situation where CBDCA causes less gastrointestinal symptomatology than CHIP but where both cause less than cis-platinum. Dexamethasone does not act by mucosal protection to provide its useful effects in prolonged cis-platinum-induced gastrointestinal symptoms.

Since its introduction in the 1970s, cis-platinum has become one of the most useful cytotoxic drugs in clinical practice. However despite this the toxicity associated with its use, *viz.* dose-limiting renal toxicity, profound gastrointestinal symptomatology and ototoxicity have been causes of great concern. Methods of circumventing the renal toxicity ensured its continued usage (Comis, 1980) but the highly emetogenic nature of the compound is a major drawback for patients receiving cancer chemotherapy. Both central mechanisms (Borison & McCarthy, 1983) and peripheral mechanisms (Akwari, 1983) are thought to be involved in the acute emesis associated with cis-platinum. The pathogenesis of the prolonged gastrointestinal symptoms, often up to 1 week after drug administration, is uncertain and small-intestinal mucosal toxicity may be involved. The development of analogues of cis-platinum was aimed at producing effective anti-tumour agents with less toxicity than the original drug. Cis-diammine-1,1-cyclobutane dicarboxylate platinum (II) [CBDCA] and cis-dichloro trans dihydroxy isopropylamine (IV) [CHIP] are two such compounds which have entered clinical trial (Calvert *et al.*, 1982; Creaven *et al.*, 1983). Neither compound is associated with significant renal toxicity and although it seems that both compounds may cause emesis which is both less acute and less prolonged than cis-platinum, CHIP does cause more gastrointestinal symptomatology than CBDCA.

Considerable improvements in the control of cis-platinum-induced emesis have been made with high dose metoclopramide being the first advance in this context (Gralla *et al.*, 1981). Recently dexamethasone has been shown to enhance significantly the efficacy of high dose metoclopramide and to reduce the duration of subsequent nausea (Allan *et al.*, 1984). The mechanism of action of dexamethasone as an anti-emetic is not known.

These studies compare the effects of CHIP and CBDCA with cis-platinum on the crypt cell production, morphology and functional activity of the mouse small intestine. In addition the effects of a combination of dexamethasone and cis-platinum on mouse small-intestinal mucosal toxicity are addressed. It was hoped that the role of gastro-

Correspondence: S.G. Allan.
Received 5 July 1985; and in revised form, 26 November 1985.

© The Macmillan Press Ltd., 1986

intestinal mucosal damage in the aetiology of cis-platinum-induced emesis would be further elucidated and that the mucosal effects of dexamethasone in combination with cis-platinum may be protective.

### Materials and methods

All platinum compounds were dissolved in water for injection i.p. into adult male CBA mice. In the comparison of the platinum analogues maximally tolerated doses of drugs were used, cis-platinum $10\,mg\,kg^{-1}$, CBDCA $100\,mg\,kg^{-1}$ and CHIP $40\,mg\,kg^{-1}$. Sections of ileum were taken on several days (1, 3, 5, 7 and 10) after injection of the drugs, in groups of four mice per drug per day. Colchicine $5\,mg\,kg^{-1}$ i.p. was administered to induce metaphase arrest in the intestinal crypts (Tannock, 1969) and one mouse from each group was sacrificed at 30 min, 60 min, 90 min and 120 min following colchicine to allow later measuring of the metaphase accumulation rate. A portion of ileum (95th per cent of small-intestinal length) from each mouse was placed in Clarke's fixative for 24 h prior to transfer to 75% ethanol for storage. A modified microdissection technique was employed, utilising Feulgen staining (Ferguson *et al.*, 1977), to measure individual villus and crypt heights, crypt to villus number ratios and crypt cell production rate (CCPR). The means of 20 measurements of villi and crypt height from each treatment group were calculated. CCPR was obtained from the rate of accumulation of blocked metaphases, counted visually and derived by linear regression based on a mean count of ten crypts per animal. Villus epithelial cell influx was derived from the product of CCPR and crypt:villus ratio. To assess small bowel function, disaccharidase activity was measured (trehalase, sucrase). A portion of jejunum (35th per cent of small-intestinal length) from each mouse was frozen and later analysed for trehalase and sucrase activity by the method of Dahlqvist (1964).

Two experiments were performed with a cis-platinum/dexamethasone combination, looking firstly at mucosal toxicity after a single exposure (acute) to the drugs, the other (chronic) observing effects after a sixth exposure at 18 weeks. In the acute dexamethasone/cis-platinum experiment groups of 6 male CBA mice were used and in the chronic experiment groups of 3. Drugs were administered to groups as follows (i) saline i.p.; (ii) dexamethasone $4\,mg\,kg^{-1}$ s/c; (iii) cis-platinum $5\,mg\,kg^{-1}$ i.p.; (iv) cis-platinum $5\,mg\,kg^{-1}$ i.p. half an hour after dexamethasone $4\,mg\,kg^{-1}$ s/c. The cis-platinum dose was designed to ensure full survival throughout the chronic experiment and the dexamethasone dose was chosen to approximate to potential human doses (Freireich, 1966). In the chronic experiment drug injections were repeated at 3-weekly intervals for 6 courses and weights were recorded weekly throughout. Metaphase arrest was achieved in the intestinal crypts using colchicine and portions of intestine taken and processed as previously described. Ten measurements of villus and crypt heights from each gut portion were made in these experiments and the metaphase count on each animal was a mean of 10 crypts as before. In view of the multiple measurements used and the variables involved, statistical analysis was by analysis of variance and then comparison of individual means by *t*-test.

### Results

In the experiment comparing platinum analogues a profound effect on CCPR was evident in all treatment groups on day 1 post injections which resulted in a marked reduction in villus epithelial cell influx. The reduction in villus influx was most marked in the CHIP treated group (Table I). Over the succeeding days an apparent loss of crypts in the CHIP and cis-platinum treated groups was noted, reducing the crypt: villus ratio, although this did not reach statistical significance. Therefore, despite evidence of an increased CCPR on days 5–10 in both these groups villus influx did not show a compensatory over production until day 7. In the CBDCA group over production of villus influx was evident on day 3. Reductions of villus height were observed at intervals following reduction in villus influx. Thus a significant reduction in villus height was seen between days 3–7 in the cis-platinum group and significantly more profound effects were noted in the CHIP group on days 1 and 3 (Figure 1). CBDCA caused a significant diminution of villus height on day 3 with recovery by day 5 although a secondary reduction in villus height was noted at day 10 which may correlate with an apparent secondary dip in villus epithelial cell influx on day 5 (Table I). The crypt heights give some indication of proliferative activity in response to the cytotoxic insult but are not discussed further.

Disaccharidase activity in jejunal mucosa was used as a functional test of effects of the platinum analogues. Both sucrase and trehalase activities were measured with both showing a similar pattern. The trehalase results are presented in Table II with a statistical comparison. Cis-platinum caused a significant reduction in trehalase activity on days 3, 5 and 7 compared with control and although CHIP produced the most profound effects on trehalase activity, recovering by day 10, these effects were not significantly different from the cis-platinum

**Table I (a)** Villus influx (ileum) – comparison of platinum analogues

| Treatment group | Day post injection 1 | 3 | 5 | 7 | 10 |
|---|---|---|---|---|---|
| *Saline controls* | | | | | |
| CCPR (cells/h) | 5.5 | '5.3 | 6.1 | 5.6 | 6.4 |
| Crypt: villus ratio | 4.9 | 4.9 | 5.0 | 4.1 | 4.8 |
| Villus influx | 27.2 | 26.0 | 31.1 | 23.7 | 31.4 |
| *Cis-platinum 10 mg kg⁻¹* | | | | | |
| CCPR (cells/h) | 2.8 | 4.1 | 6.8 | 10.8 | 9.4 |
| Crypt: villus ratio | 3.8 | 3.3 | 4.2 | 3.5 | 4.7 |
| Villus influx | 10.9 | 13.6 | 29.0 | 38.0 | 44.8 |
| *CBDCA (100 mg kg⁻¹)* | | | | | |
| CCPR (cells/h) | 2.4 | 10.2 | 4.6 | 11.3 | 6.5 |
| Crypt: villus ratio | 4.5 | 5.0 | 5.0 | 4.8 | 4.9 |
| Villus influx | 11.0 | 51.5 | 23.7 | 54.8 | 32.1 |
| *CHIP (40 mg kg⁻¹)* | | | | | |
| CCPR (cells/h) | 0.4 | 2.9 | 11.2 | 18.6 | 8.1 |
| Crypt: villus ratio | 3.2 | 3.0 | 2.2 | 2.5 | 3.3 |
| Villus influx | 1.3 | 8.9 | 25.6 | 47.1 | 27.1 |

**Table I (b)** Statistical comparison[a] of villus influx-platinum analogues

| Comparison | Day post injection 1 | 3 | 5 | 7 | 10 |
|---|---|---|---|---|---|
| [b]cP *vs.* CBDCA | NS | <0.001 | NS | NS | <0.025 |
| [b]cP *vs.* CHIP | <0.001 | NS | NS | NS | <0.05 |
| CBDCA *vs.* CHIP | <0.005 | <0.001 | NS | NS | NS |

[a]$P$ values by analysis of variance ($t$ test on mean values). [b]cP = cis-platinum.

**Figure 1** Comparison of effects of platinate compounds on villus and crypt heights. Each bar is the mean of 20 villi or crypts from 4 mice (cis-platinum 10 mg kg⁻¹: CBDCA 100 mg kg⁻¹: CHIP 40 mg kg⁻¹). Control (□); CBDCA (▨); cis-platinum (▩); CHIP (■). * Significant difference from saline control ($P<0.005$); + Significant difference from CP control ($P<0.05$).

**Table II** Comparison of trehalase activity[a] following platinum analogues

| | Day post injection 1 | 3 | 5 | 7 | 10 |
|---|---|---|---|---|---|
| Control | 15.5 | 13.9 | 13.9 | 13.7 | 16.5 |
| Cis-platinum | 15.9 | 6.4 | 6.8 | 9.3 | 23.9 |
| CBDCA | 9.2 | 10.2 | 11.2 | 12.2 | 17.2 |
| CHIP | 15.4 | 8.2 | 4.4 | 7.3 | 16.9 |

[a]Activity = $\mu$mol substrate $min^{-1} g^{-1}$ wet wt.

Difference significant, $P<0.025$ (analysis of variance):

| | | |
|---|---|---|
| Cis-platinum | *vs.* Control | Day 3, 5, 7, 10 |
| Cis-platinum | *vs.* CBDCA | Day 1, 3, 5, 10 |
| Cis-platinum | *vs.* CHIP | N.S. |
| CBDCA | *vs.* Control | Day 1, 3 |
| CBDCA | *vs.* CHIP | Day 1, 5, 7 |
| CHIP | *vs.* Control | Day 3, 5, 7 |

group. CBDCA again showed least effect and no significant difference from control values was present by day 5. A rather high value was obtained in the cis-platinum group on day 10.

In the experiments with dexamethasone cis-platinum 5 mg/kg causes marked inhibition of CCPR as seen 24 hours after injection but crypt regeneration is evident by day 3 as demonstrated by an increased CCPR and villus epithelial cell influx (Table III). Consequent to the reduction in villus influx the villus height is maximally diminished by day 3 but recovers thereafter (Figure 2). At this dose of cis-platinum no effect was seen on the crypt to villus ratio and thus the changes in villus influx were mainly dependent on CCPR. No significant kinetic or morphological change was noted in the dexamethasone controls and when dexamethasone was combined with cis-platinum it was clear that no protective effect is afforded to the ileum with this combination (Table III and Figure 2). Indeed the mean villus height is significantly less ($P<0.02$) in the combination treated group compared with the cis-platinum group on days 5, 7 and 10 although no significant differences were calculated in villus influx.

**Table III** Villus influx (ileum) – cis-platinum and dexamethasone (single injections)

| Treatment group | Day post injection 1 | 3 | 5 | 7 | 10 |
|---|---|---|---|---|---|
| *Saline controls* | | | | | |
| CCPR (cells/h) | 6.0 | 6.1 | 5.8 | 5.6 | 5.5 |
| Crypt: villus ratio | 4.9 | 4.9 | 5.6 | 5.0 | 4.9 |
| Villus influx | 30.1 | 30.8 | 33.1 | 28.5 | 27.3 |
| *Dexamethasone controls* | | | | | |
| CCPR (cells/h) | 7.2 | 7.3 | 5.9 | 5.4 | 5.7 |
| Crypt: villus ratio | 4.6 | 5.2 | 4.9 | 4.8 | 5.1 |
| Villus influx | 34.1 | 38.8 | 29.4 | 26.2 | 29.3 |
| *Cis-platinum* | | | | | |
| CCPR (cells/h) | 1.6 | 10.1 | 6.8 | 8.1 | 6.5 |
| Crypt: villus ratio | 4.2 | 4.4 | 4.3 | 5.2 | 4.7 |
| Villus influx | 7.0[a] | 45.5[a] | 30.0 | 42.6[a] | 30.8[b] |
| *Cis-platinum/dexamethasone* | | | | | |
| CCPR (cells/h) | 1.9 | 8.4 | 8.6 | 6.9 | 8.1 |
| Crypt: villus ratio | 4.6 | 4.0 | 4.5 | 5.1 | 5.0 |
| Villus influx | 9.1[a] | 34.5 | 39.5 | 35.5 | 41.2[ab] |

[a]Significant difference from control values, $P<0.05$ (analysis of variance). [b]Significant difference between groups 3 and 4, $P<0.05$ (analysis of variance).

In the experiment involving chronic administration of cis-platinum and dexamethasone the pattern of response of the ileum to the cytotoxic insult of cis-platinum was similar to that seen in the acute situation (Table IV). However, the combination of dexamethasone and cis-platinum in chronic dosing when compared with the cis-platinum group demonstrated a greater reduction in villus influx on day 1 ($P<0.05$) and a slower recovery on day 7 ($P<0.05$). The administration of dexamethasone to control mice did not alter significantly their weights over an 18 week period compared with saline controls. Both the cis-platinum receiving groups failed to gain weight and differed significantly from the control groups throughout the observation period ($P<0.05$ analysis of variance) but did not differ from each other (Figure 3).

### Discussion

The integrity of the intestinal mucosa is dependent on the crypt cell production rate and the crypt: villus ratio. The product of these, the epithelial cell influx can be profoundly influenced by changes in the crypt cell production rate and the number of residual crypts. Cis-platinum causes a profound reduction in CCPR, maximal between 12–24 h (unpublished data) with a period of compensatory rebound production which timing is dependent on the cis-platinum dose administered. At higher doses

**Figure 2** Comparison of villus and crypt heights following a cis-platinum/dexamethasone combination. Each bar is the mean of 60 villi or crypts from 6 mice (cis-platinum 5 mg kg$^{-1}$: dexamethasone 4 mg kg$^{-1}$ [acute]). Saline control (□); dexamethasone control (▩); cis-platinum (▧); cis-platinum + dexamethasone (■). *, difference between CP and CP/dex ($P<0.02$, ANOVA).

**Figure 3** Weight changes following chronic intermittent cis-platinum/dexamethasone dosing (cis-platinum 5 mg kg$^{-1}$ *q* 3 weekly: dexamethasone 4 mg kg$^{-1}$ *q* 3 weekly). Saline control (○); dexamethasone controls (□); platinum (●—●); platinum + dexamethasone (●---●). Each point is the mean of 12 mice.

**Table IV** Villus influx (ileum) – cis-platinum and dexamethasone (chronic dosing)

| | Day post injection | | | |
|---|---|---|---|---|
| *Treatment group* | *1* | *3* | *5* | *7* |
| *Saline controls* | | | | |
| CCPR (cells/h) | 5.8 | 5.7 | 5.6 | 6.3 |
| Crypt: villus ratio | 5.4 | 4.8 | 5.0 | 5.1 |
| Villus influx | 31.5 | 27.7 | 28.5 | 32.9 |
| *Dexamethasone controls* | | | | |
| CCPR (cells/h) | 6.2 | 5.6 | 5.7 | 7.1 |
| Crypt: villus ratio | 5.1 | 4.2 | 4.6 | 5.4 |
| Villus influx | 32.2 | 24.3 | 26.8 | 39.0 |
| *Cis-platinum* | | | | |
| CCPR (cells/h) | 1.7 | 14.6 | 7.9 | 8.4 |
| Crypt: villus ratio | 4.9 | 4.1 | 5.6 | 4.9 |
| Villus influx | 8.8[a] | 60.7[a] | 44.8[a] | 41.6[b] |
| *Cis-platinum/dexamethasone* | | | | |
| CCPR (cells/h) | 1.0 | 11.8 | 7.5 | 6.1 |
| Crypt: villus ratio | 4.1 | 4.0 | 4.6 | 4.9 |
| Villus influx | 4.3[a] | 47.5[a] | 35.11 | 30.7[b] |

[a]Significant difference from control, $P<0.05$. [b]Groups 3 and 4 significantly different $P<0.05$ (analysis of variance).

of cis-platinum an additional, smaller effect on villus influx is provided by the temporary ablation of crypts, although this loss was not statistically significant. Following these effects on villus influx the villus height becomes stunted and mucosal function, as measured here by disaccharidase activity, is diminished. When cis-platinum $5\,mg\,kg^{-1}$ was administered in a chronic intermittent fashion the pattern of mucosal toxicity and recovery was similar to that seen in the acute situation, suggesting that crypt tolerance to the repeated cytotoxic insult of cis-platinum is high.

When the platinum analogues are compared with cis-platinum, CHIP appears to be at least as toxic if not more toxic to the intestinal mucosa with compensatory rebound in villus influx occurring only by day 7. CBDCA was the least toxic and recovery was much more rapid although a secondary unexplained reduction of villus height was noted on day 10. CHIP and cis-platinum appeared to cause loss of crypts but because of the small sample sizes this did not reach statistical significance. CBDCA caused least depression of disaccharidase activity with recovery by day 5, whereas CHIP and cis-platinum caused prolonged depression beyond 7 days. The isolated high trehalase value for cis-platinum is unexplained but was not observed in estimations of sucrase activity (unpublished data). It is clear from early clinical studies of CHIP and CBDCA that gastrointestinal toxicity, both acute and chronic emesis and diarrhoea, is less than that produced by the parent compound with CBDCA the least offensive. In the mouse, CBDCA produces the least mucosal toxicity of the three compounds examined but as CHIP is as toxic as cis-platinum in the above experiments no clear inference can be drawn with respect to the human situation. It is clear that cis-platinum and CHIP produce effects on the intestinal mucosa which last for many days after the cytotoxic insult and in the case of the former, control values of CCPR are reached only by day 12 (unpublished data). The correlation of prolonged gastrointestinal symptoms with intestinal mucosal toxicity in patients receiving platinate compounds is unproven and human intestinal biopsies, in patients receiving these compounds, may help in solving this issue. Where efficacy is established the use of these platinum analogues will reduce the morbidity of cancer chemotherapy but where cis-platinum continues to be used then amelioration of associated gastrointestinal symptoms may be achieved by effective anti-emesis (Allan *et al.*, 1984) or potentially by antidoting cis-platinum intestinal cytotoxicity (Allan *et al.*, 1985).

The useful anti-emetic activity of dexamethasone both in non-cis-platinum and cis-platinum containing chemotherapy-induced vomiting (Cassileth *et al.*, 1983; Allan *et al.*, 1984) is unexplained. The latter study showed that dexamethasone could shorten the prolonged nausea associated with cis-platinum administration. The dexamethasone/cis-platinum experiments addressed the question of whether dexamethasone had some protective role on cis-platinum-induced mucosal toxicity in the mouse. Clearly dexamethasone does not act to prevent this toxicity and indeed it may enhance cis-platinum toxicity. Although it has been reported that glucocorticoids can stimulate intestinal mucosal proliferation (Eastwood *et al.*, 1981) the balance of evidence is probably to the contrary. Wright & Alison (1984) argue from the literature that glucocorticoids cause a block in $G_{1-S}$ transition in the cell cycle. The addition of dexamethasone does not prevent the weight loss associated with cis-platinum in chronic dosing in the mouse. In man dexamethasone effectively reduces cis-platinum associated gastrointestinal symptoms but it may well exert its effects centrally both to control the acute emesis and the subsequent prolonged nausea, vomiting and anorexia resulting from cis-platinum. Dexamethasone may act peripherally to enhance appetite following cis-platinum but in the mouse does not protect against structural mucosal toxicity and thus will probably not alter the digestive and absorbtive impairment which may ensue.

The role of small-intestinal mucosal toxicity in the pathogenesis of prolonged gastrointestinal symptoms subsequent to the use of cis-platinum remains uncertain. Platinum analogues produce intestinal toxicity in the mouse with varying intensity. No direct inference can be drawn in regard to the degree of murine mucosal toxicity and the degree of gastrointestinal toxicity induced by platinate compounds in the clinical situation. Dexamethasone does not protect the small-intestinal mucosa from the effects of cis-platinum.

**References**

AKWARI, O.E. (1983). The gastrointestinal tract in chemotherapy-induced emesis – a final common pathway. *Drugs*, **25** (suppl. 1), 18.

ALLAN, S.G., CORNBLEET, M.A., WARRINGTON, P.S., GOLLAND, I.M., LEONARD, R.F.C. & SMYTH, J.F. (1984). Dexamethasone and high dose metoclopramide – efficacy in cis-platinum-induced emesis. *Br. Med. J.*, **289,** 878.

ALLAN, S.G., HAY, F.G., LEONARD, R.C.F., SMYTH, J.F. & WOLF, C.R. (1985). Protective effect of mesna on the gastrointestinal toxicity of cis-platinum. *Br. J. Cancer,* **52,** 454 (abstract).

BORISON, H.L. & McCARTHY, L.E. (1983). Neuropharmacology of chemotherapy-induced emesis. *Drugs*, **25** (suppl. 1), 8.

CALVERT, A.H.,HARLAND, S.J., NEWELL, D.R. & 9 others (1982). Early clinical trials with cis-diammine-1, 1-cyclobutane dicarboxylate platinum (II). *Cancer Chemother. Pharmacol.*, **9,** 140.

CASSILETH, P.A., LUSK, E.J., TORRI, S., DINUBLE, N. & GERSON, S.L. (1983). Anti-emetic efficacy of dexamethasone therapy in patients receiving cancer chemotherapy. *Arch Intern Med.*, **143,** 1347.

COMIS, R.L. (1980) Cis-platinum nephrotoxicity. In *Cis-platin – Current Status and New Developments*, Prestayko, Crooke & Carter (eds) *p*. 485. Academic Press Inc: New York.

CREAVEN, P.J., MADAJEWICZ, S., PENDYALA, L. & 5 others (1983). Phase I clinical trial of cis-dichloro-trans-dihydroxy-bis-isopropylamine platinum IV (CHIP). *Cancer Treat Rep.*, **67,** 795.

DAHLQVIST, A. (1964). Method for assay of intestinal disaccharidases. *Analyt. Biochem.*, **7,** 18.

EASTWOOD, G.L., QUIMBY, G.P. & LAFERRIERE, J.R. (1981). Effects of chronic steroid ingestion on gastro-duodenal epithelial renewal in the rat. *Cell Tissue Kinet*, **14,** 405.

FERGUSON, A., SUTHERLAND, A., MacDONALD, T.T. & ALLAN, F. (1977). Technique for microdissection and measurement in biopsies of human small intestine. *J. Clin. Path.*, **30,** 1068.

FREIREICH, E.J., GEHAN, E.A., RALL, D.P., SCHMIDT, L.H. & SKIPPER, H.E. (1966). Quantitative comparison of toxicity of anti-cancer agents in mouse, rat, hamster, dog, monkey and man. *Cancer Chemother Rep.*, **50,** 219.

GRALLA, R.J., ITRI, L.M., PISKO, S.E. & 6 others (1981). Anti-emetic efficacy of high dose metoclopramide. *New Engl. J. Med.*, **305,** 905.

TANNOCK, I.F. (1969). A comparison of the relative efficiencies of various metaphase arrest agents. *Exp. Cell Res.*, **47,** 345.

WRIGHT, N. & ALISON, M. (1984). Growth and proliferative changes in the gastrointestinal tract. In *The Biology of Epithelial Cell Populations*, Wright & Alison (eds) **2,** p. 775. Clarendon Press: Oxford.